# CD40 Ligand and Autoantigen Are Involved in the Pathogenesis of Low-Grade B-Cell Lymphomas of Mucosa-Associated Lymphoid Tissue

**DOI:** 10.1155/1998/18679

**Published:** 1998

**Authors:** A. Greiner, C. Knörr, Y. Qin, A. Schultz, A. Marx, R. A. Kroczek, H. K. Müller-Hermelink

**Affiliations:** 1 Institute of Pathology University of Würzburg Germany; 2 Robert-Koch-Institut Berlin Germany; 3 Institut für Pathologie der Univerisitãt Würzburg Josef-Schneider str. 2 Würzburg 97080 Germany

**Keywords:** Lymphoma, hypermutation, CD40L, autoantigen, idiotype, antigen receptor

## Abstract

Low-grade MALT-type lymphomas are malignancies of mucosal marginal-zone B cells and preceded by reactive inflammatory lymphoid tissue. Experimental observations suggest that antigen and CD40 Ligand act during cognate T/B cell interaction and are crucial for germinal center B-cell maturation generating marginal-zone B cells. To investigate the mechanisms underlying the development of extranodal MALT-type lymphomas, the immunoglobulin receptor was sequenced and analyzed for antigen specificity using heterohybridoma technology. Furthermore, CD40 ligand expression was evaluated by immunohistochemistry and by semiquantitative RT-PCR, and ligand binding to the CD40 of tumor B cells was studied using the CD40 system. Hypermutations were found in low-grade lymphomas throughout CDR1- CDR3 suggestive of positive selection through their antigen receptor. Different VH families were used and more than 69% of tumor immunoglobulins bound different mucosal antigens. CD40L expression was found in the tumor marginal zone in substantial amounts. The *in vitro* proliferation response of all low-grade MALT-type lymphomas was dependent on anti-CD40- mediated signals and cytokines. Our data provide evidence that autoantigen as well as the CD40L expressed by activated nonneoplastic T cells may drive the evolution of low-grade MALT-type lymphomas either directly or by paracrine mechanisms and that antigen may contribute to lymphoma pathogenesis.


*Developmental Immunology*, 1998, Vol. 6, pp. 187–196
Reprints available directly from the publisher
Photocopying permitted by license only

© 1998 OPA (Overseas Publishers Association)
N.V. Published by license under the
Harwood Academic Publishers imprint,
part of the Gordon and Breach Publishing Group
Printed in Malaysia

### CD40 Ligand and Autoantigen Are Involved in the Pathogenesis of Low-Grade B-Cell Lymphomas of Mucosa-Associated Lymphoid Tissue

A. GREINER[a,*], C. KNÖRR[a], Y. QIN[a], A. SCHULTZ[a], A. MARX[a], R. A. KROCZEK[b] and H. K. MÜLLER-HERMELINK[b]

[a]*Institute of Pathology, University of Würzburg*; [b]*Robert-Koch-Institut, Berlin, Germany*

*(Received 12 October 1996)*

Low-grade MALT-type lymphomas are malignancies of mucosal marginal-zone B cells and preceded by reactive inflammatory lymphoid tissue. Experimental observations suggest that antigen and CD40 Ligand act during cognate T/B cell interaction and are crucial for germinal center B-cell maturation generating marginal-zone B cells. To investigate the mechanisms underlying the development of extranodal MALT-type lymphomas, the immunoglobulin receptor was sequenced and analyzed for antigen specificity using heterohybridoma technology. Furthermore, CD40 ligand expression was evaluated by immunohistochemistry and by semiquantitative RT-PCR, and ligand binding to the CD40 of tumor B cells was studied using the CD40 system. Hypermutations were found in low-grade lymphomas throughout CDR1-CDR3 suggestive of positive selection through their antigen receptor. Different VH families were used and more than 69% of tumor immunoglobulins bound different mucosal antigens. CD40L expression was found in the tumor marginal zone in substantial amounts. The *in vitro* proliferation response of all low-grade MALT-type lymphomas was dependent on anti-CD40-mediated signals and cytokines. Our data provide evidence that autoantigen as well as the CD40L expressed by activated nonneoplastic T cells may drive the evolution of low-grade MALT-type lymphomas either directly or by paracrine mechanisms and that antigen may contribute to lymphoma pathogenesis.

*Keywords:* Lymphoma, hypermutation, CD40L, autoantigen, idiotype, antigen receptor

### INTRODUCTION

More than one-third of non-Hodgkin lymphomas (NHL) arise at sites other than lymph nodes, the so-called extranodal lymphomas (Isaacson and Spencer, 1987). They are almost all of B cell origin and emerge preferentially in the stomach, an organ primarily devoid of preexisting mucosa-associated lymphoid

*Corresponding author. Present address. Institut für Pathologie der Univerisität Würzburg, Josef-Schneider str. 2, 97080 Würzburg, Germany

TABLE I VH Gene Analysis of Low-Grade MALT-Type Lymphoma with a Pattern of Somatic Mutations

| Case | ClosestVH member | | Identity | No. of nucleotide difference (total in) | | | | | | | R:S | |
|---|---|---|---|---|---|---|---|---|---|---|---|---|
| | VHgene family | Germ-line genomic | Identity | FR1 | CDR1 | FR2 | CDR2 | FR3 | (CDRs | FRs) | CDRs | FRs |
| 1 | VHI | hv1263 | 94% | 1 | 2 | 1 | 4 | 9 | 6 | 11 | 6.0 | 0.8 |
| 2 | VHI | hv35 | 95% | 3 | 1 | 0 | 5 | 5 | 6 | 8 | 6.0 | 6.0 |
| 3 | VHIII | hv3005 | 95% | 4 | 2 | 1 | 4 | 3 | 7 | 7 | 4.0 | 0.4 |
| 4 | VHIV | VH4-21 | 94% | 3 | 4 | 2 | 2 | 5 | 6 | 10 | 4.0 | 1.25 |

tissue (MALT). Highly organized lymphoid tissue of MALT-type, however, is formed as a consequence of chronic inflammation. Since morphological and functional features of this lymphoma are similar to primary MALT, it has been designated secondary MALT (Greiner and Müller-Hermelink, 1996). The conditions leading to secondary MALT are considered as important preconditions of lymphomagenesis at extranodal sites (Isaacson, 1994).

It has been suggested that marginal-zone B cells are the normal counterpart to MALT-type lymphoma tumor cells. The former are noncirculating memory B cells and generated during T cell-dependent antigen responses (Dunn Walters et al. 1995). Experimental observations suggest 'that antigen and CD40/CD40 ligand (T-BAM, TRAP) act during cognate T/B cell interaction and are crucial checkpoints for germinal-center B cell maturation generating marginal-zone B cells (Arpin et al., 1995; Han et al., 1995). The maturation of B cells into efficient producers of high-affinity and high-specificity antibodies is regulated by various T cell subsets through cell-surface molecules and soluble cytokines (Banchereau et al., 1994). In particular, IL-2, IL-4, IL-6, and IL-10 have been demonstrated to be crucial for B cell maturation (Fluckiger et al., 1993; Briere et al., 1994; Burdin et al., 1995).

Therefore, T cells may determine not only the type of B cell immune response but may also provide key molecules in B cell lymphoma initiation and progression as well. Both, the histopathological and clinical features of MALT-type lymphomas suggest the possibility that the lymphomagenesis is, at least in part, antigen-dependent.

### RESULTS

### Tumor Antigen Receptor Is Hypermutated and Recognizes Autoantigen

Analysis of the VH regions of MALT lymphomas showed usage of different VH families (Table I) (Qin et al., 1995). Additionally, compared with the germline, the VH gene of each tumor contained at least 14 substitutions. They were distributed in a pattern characteristic for an antigen-driven affinity maturation, that is, the somatic mutations were highly concentrated in the CDRs and FRs with a clustering of replacement [R] mutations in the CDRs, but only few in the FRs. Three of the four genes detected here, hv1263, hv3005, and VH4-21, are frequently used in a variety of autoantibodies, such as cold agglutinins, rheumatoid factors and anti-DNA antibodies (Dersimonian et al., 1987; Sanz et al., 1989; Olee et al., 1991).

### Analysis of the Tumor Immunoglobulin Specificity

The antibody specificity of monoclonal antibodies derived from 13 low- and high-grade MALT-type lymphomas of the stomach, thyroid, salivary gland, and the lung were analyzed by using heterohybridoma technology. Tumor immunoglobulin from 9 of 13 patients (69%) had autoantibody activity (Table II). The target antigens of the lymphoma immunoglobulin have been found to be autoantigens mainly expressed in mucosal tissues, for example, of thyroid, parotid, lung, or stomach tissue. The human Abs bound to different self-determinants and fullfilled the definition

TABLE II Tumor Immunoglobulin Specificity on Human Cryostat Sections

| Lymphoma | Site of origin | Binding specificity |
|---|---|---|
| 1 | Stomach | ANA, salivary gland, thyroid epithelium |
| 2 | Stomach | — |
| 3 | Stomach | — |
| 4 | Stomach | Small blood vessels |
| 5 | Stomach | Salivary gland, foveolar epithelium in the stomach |
| 6 | Stomach | Salivary gland, thyroid epithelium |
| 7 | Stomach | Lung epithelium |
| 8 | Stomach | Lung epithelium |
| 9 | Stomach | Mucosal plasma cells |
| 10 | Stomach | — |
| 11 | Lung | ANA, salivary gland, thyroid epithelium, glandular epithelium in the stomach |
| 12 | Lung | — |
| 13 | Thyroid | Thyreoglobulin |

of an autoantibody since they are self-reactive, but not self-specific. These monoclonal antibodies (mAbs) were highly specific for the identified antigen and bound none of the other tested antigens that are commonly recognized by polyreactive natural autoantibodies (e.g., rheumatoid factor, myosin, and actin).

### The Tumor Antigen Receptor Is Functionally Active

By using an anti-idiotypic antibody highly specific for a gastric IgA-expressing low-grade MALT lymphoma immunoglobulin receptor (idiotype), induction of the proliferation response after cross-linking was induced by additional stimulation with a mitogen. A specific growth promotion effect of the anti-idiotype antibody in combination with SAC was noted in the lymphoma cells but not in controls. The effect of the anti-idiotype antibody induced proliferation was more pronounced than the effect of cytokine or mitogen stimulation alone (Figure 1A). This may indicate a dependency on additional signals. Furthermore, stimulation with the anti-idiotype antibody induced a differentiation of tumor cells, as demonstrated by an enhanced secretion of tumor IgA (Fig. 1b), but not of control IgM (Fig. 1c).

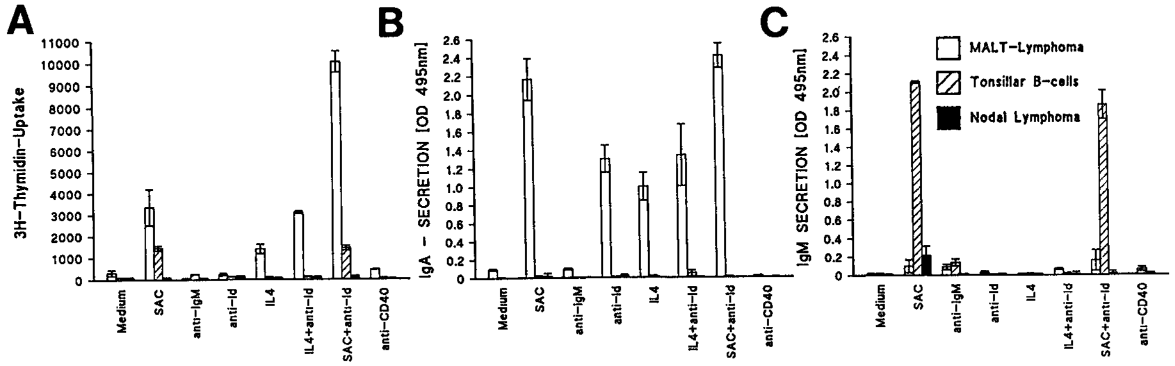

Fig. 1 Stimulation of purified tumor B cells of IgA-expressing MALT-type lymphoma and follicular nodal lymphoma as neoplastic and tonsilar as non-neoplastic B cells as control. (A) $5 \times 10^4$ purified B cells were cultured in triplicate over 5 days. [3H]TdR uptake was measured after a 16-hr pulse. Note the enhanced proliferation of MALT tumor B cells but not of controls using the anti-Id antibody. (B) Differentiation of MALT tumor B cells using the anti-Id antibody as demonstrated by IgA-secretion. This effect of the anti-idiotype antibody was found only in the tumor, but not in tonsilar or nodal tumor B cells. Within the latter, only SAC was able to induce differentiation as demonstrated by IgM-secretion (C).

### CD40 Receptor Is Expressed by Tumor B Cells and CD40 Ligand Is Present in Tumor Tissue

All investigated and freshly isolated MALT lymphoma B cells expressed high levels of CD40 on their cell surfaces (Fig. 2). Nontumor T cells expressing CD40L were found within the tumor marginal zone by *in situ* immunohistochemistry. By using a semiquantitative RT-PCR, CD40L was found to be increased in tumor tissues of low-grade MALT-type lymphomas in contrast to controls (reactive, nonneoplastic tissues) (Fig. 3).

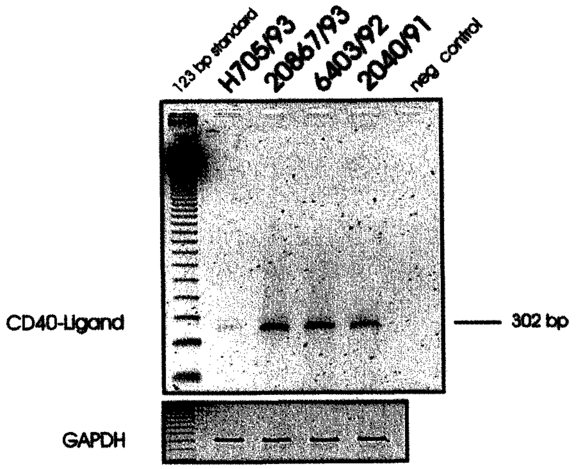

Fig. 3 Semiquantitative RT-PCR using GADPH as a standard showing CD40L message in cases of low-grade MALT-type B cell lymphomas.

### Functional Consequences of Antigen Receptor and CD40 Engagement in MALT Lymphoma

To determine whether the tumor cell response in low-grade lymphoma is due to a stimulatory effect of antigen or due to effects solely mediated by tumor-infiltrating T cells the B cells were cultured in the so-called "CD40-system," which allowed to study the properties of tumor B cells and nontumor T cells independently (Fig. 4). Herein the proliferative B cell response of all low-grade MALT-type lymphomas

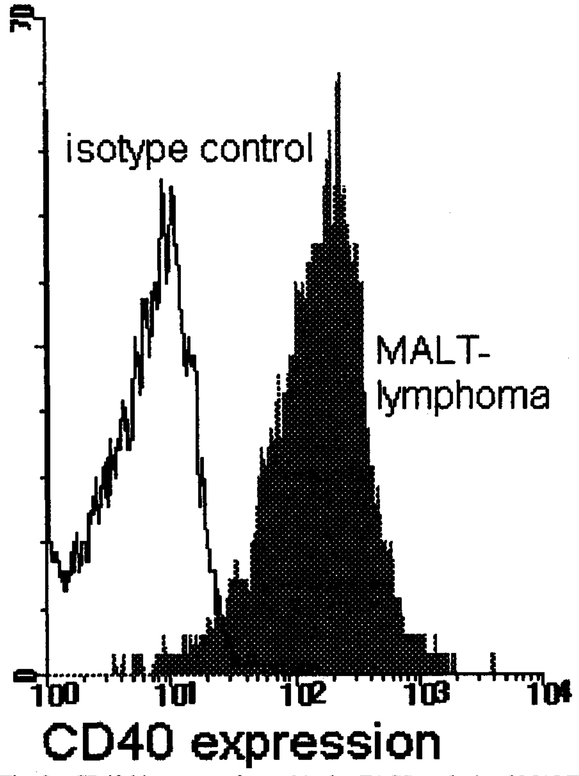

Fig. 2 CD40 histogram of a multicolor FACS analysis of MALT lymphoma B cell suspension freshly isolated after surgery and gated for viable lymphocytes, $CD19^+$, $CD40^+$, and 7-AAD, as described (Schmid et al., 1992).

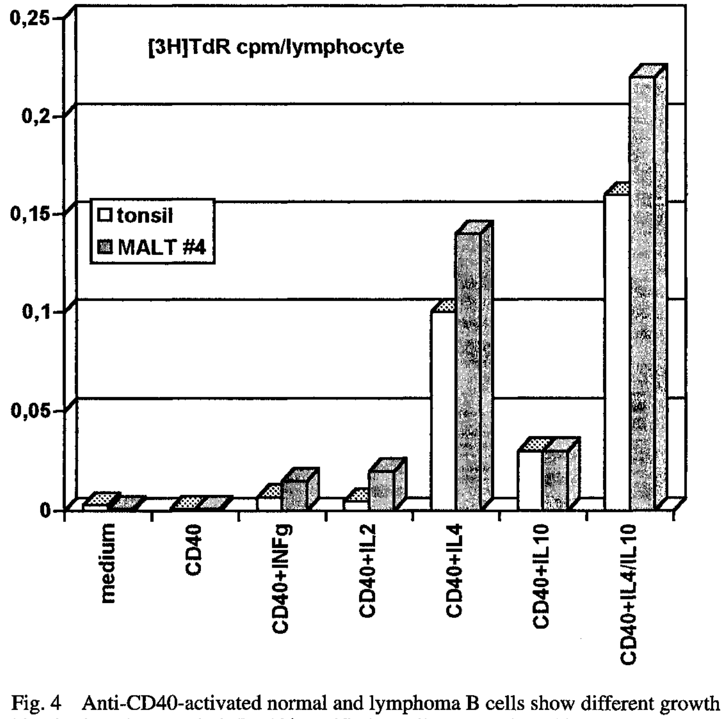

Fig. 4 Anti-CD40-activated normal and lymphoma B cells show different growth kinetics in culture period. 5 X$10^4$ purified B cells were cultured in triplicate over 5 days. [3H]T dR upta are representative of three independent experiments.

TABLE III Differentiation of Anti-CD40 Activated MALT-Type Lymphoma B Cells Is Induced by IL-10[a]

| | IgM production ($\mu$g/ml) | | | | |
|---|---|---|---|---|---|
| | Tonsil B cells | Low-Grade MALT | | | |
| | (average) | #1 | #2 | #3 | #4 |
| Medium | <0.05 | <0.05 | <0.05 | <0.05 | <0.05 |
| IL-2 | <0.05 | <0.05 | <0.05 | <0.05 | <0.05 |
| IL-4 | <0.05 | <0.05 | <0.05 | <0.05 | <0.05 |
| IL-10 | 0.18 | 7.34 | 6.68 | 10.23 | 4.22 |
| IL-10 + IL-2 | 0.42 | 7.22 | 6.99 | 9.58 | 4.45 |
| IL-10 + IL-4 | 0.22 | 7.28 | 7.29 | 10.63 | 4.87 |

[a]$5 \times 10^4$ B cells were cultured during 5 days in the CD40 system. Antibody production was measured by ELISA.

investigated was strongly dependent on anti-CD40-mediated signals, complemented by cytokines produced by T helper cells of the Th2 type (IL-4 and/or IL-10). Thl cytokines (IL-2 and/or INF$\gamma$) had little effect. No difference to B cells isolated from normal tonsils was detected. Furthermore, low-grade MALT-type lymphoma B cells were induced to secrete large amounts of tumor immunoglobulin in response to IL-10 (Table III). This antibody secretion by tumor B cells was significantly more pronounced than in normal B cells and could not be enhanced by adding IL-2 or IL-4 alone or in combination.

In contrast to CD40 ligand signaling, B-cell-receptor (BCR) triggering induced spontaneous cell death in normal tonsil but not in MALT lymphoma B cells *in vitro* (Fig. 5) reminiscent of apoptosis of germinal-center B cells *in vivo*.

### DISCUSSION

To get an insight into the pathogenesis of MALT-type lymphomas, it was the aim of the present study to investigate the eventual correlation between two observations made independently with B cell lymphomas in general and MALT lymphomas in particular: The first observation concerns the high frequency of "anti-self" or autoimmune reactivity of immunoglobulins secreted both by nodal lymphomas like CLL and follicular B cell lymphomas as well as by lymphomas of MALT (Hussell et al., 1993; Greiner et al., 1994a). The second observation made only in MALT lymphoma is that they are preceded by reactive inflammatory tissue. So far, however, it has not been defined whether antigen and paracrine factors contribute to lymphoma development.

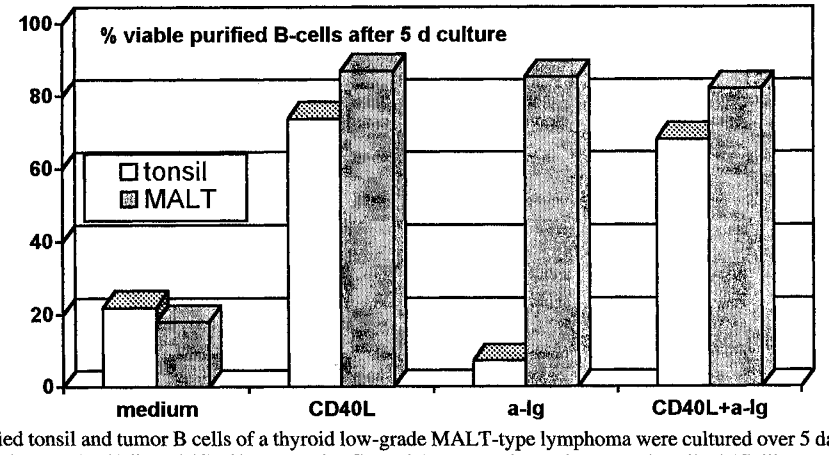

Fig. 5 Highly purified tonsil and tumor B cells of a thyroid low-grade MALT-type lymphoma were cultured over 5 days either with medium alone or recombinant human CD40 ligand (CD40L) or anti-BCR (a-Ig) as an antigen trigger, as described (Galibert et al., 1996). Viable cells were visualized using Trypan blue staining. Note a marked decrease of tonsil but not tumor B cells using a-Ig.

In particular, recent work about CD5$^+$ chronic lymphatic leukemia (B-CLL) (Schroeder, Jr. and Dighiero, 1994) or CD5$^-$ follicular lymphoma (Kobayashi et al., 1993) has provided strong evidence that a high percentage of B cells that express immunoglobulin with reactivity against self-determinants are frequently committed to malignancy. In contrast to MALT lymphoma, a restricted repertoire of immunodominant epitopes on the target antigen(s) of the lymphoma immunoglobulin (idiotype) is displayed in CLL, follicular and mantle cell lymphoma, as exemplified by a high frequency of shared idiotypes within these tumors (Dighiero, 1992; Rudders et al. 1992; Ohno et al., 1995). Consequently, the immunoglobulins of MALT-type lymphomas exhibit a highly, restricted specificity for a variety of distinctive (auto)antigens confined to MALT (e.g., mucosal plasma cells, thyroid, salivary gland, lung and stomach epithelia) that does not share idiotop within extranodal or nodal lymphomas (Greiner et al. 1994a).

This may be explained at the molecular level by the finding that MALT-lymphoma immunoglobulin gene sequences were found frequently in a variety of autoantibodies. They displayed somatic hypermutations, indicating affinity maturation during a specific antigen response that may alter the binding to the initiating antigen thus leading to a variety of different and highly specific autoantibodies (Schroder et al., 1996). The putative antigen binding to MALT-tumor immunoglobulin is still unknown and could be an exogenous antigen, a regulatory $\alpha$-idiotype antibody or an autoantigen. In this regard, experimental data favor the latter possibility.

Autoreactivity in MALT-type lymphoma may have consequences in at least two ways. First, it emphasizes the recent hypothesis that proliferation in some MALT-type lymphomas may be antigen-driven, and autoimmunity may play a role in their pathogenesis (Müller-Hermelink et al., 1995). This hypothesis is strengthened by the observation that the antigen receptor of MALT lymphomas is functionally active and can induce tumor-cell survival (Fig. 5). Second, according to the concept of the recently proposed REAL classification (Harris et al., 1994), the immunological and molecular data have helped to define MALT-type lymphoma with respect to their extraordinary pathophysiological features.

Whether antigen-driven affinity maturation is associated with secondary events that cause tumor transformation in MALT Lymphoma is uncertain. As in most nodal lymphomas, the etiology and pathogenesis of lymphomas derived from MALT are not yet elucidated. In particular, unifying chromosomal aberration (Roblick et al., 1993; Wotherspoon et al., 1995), rearrangements of oncogenes (Marin et al. 1995), or Epstein-Barr virus infections (Ott et al., 1993; Greiner et al., in press), thought to be first steps in the development of nodal lymphomas, have not been detected in lymphomas of MALT type. It is possible that antigen is necessary for survival of the initiating malignant tumor clone in combination with paracrine factors supported by reactive tumor infiltrating T cells that express CD40 ligand. In this regard, it was important that large amounts of CD40L were detected in MALT lymphoma tissues *in vivo* and that the tumor B cells responded to the CD40 ligand signal *in vitro*. The activation of the malignant B cells parallels the activation of normal mature B cells resulting in B-cell proliferation and differentiation depended on the added cytokine. In contrast, CD40 cross-linking was found to inhibit cell proliferation in other lymphoma types, like Burkitt lymphoma, diffuse large-cell lymphoma, or lymphoblastoid cell lines (Funakoshi et al., 1994).

Therefore, our data provide certain evidence that autoantigen as well as the CD40L expressed by activated non-neoplastic T cells may trigger evolution of low-grade MALT-type lymphomas either directly or by paracrine mechanisms and that antigen may contribute to lymphoma pathogenesis.

### MATERIAL AND METHODS

### Tissues, Cell Culture, and B-Cell Purification

Non-neoplastic tonsillar tissues and malignant lymphomas were obtained from biopsies after surgical removal for the preparation of cell suspensions, snap-freezing in liquid nitrogen, and routine fixation for histological examination. In all cases, the diagnosis was confirmed by morphological and immunophenotypical analysis of fresh-frozen and paraffin-embedded sections. Mononuclear single-cell suspensions were isolated by density-gradient centrifugation and negatively depleted with magnetic beads coupled either with anti-CD2, -CD14 mAbs (Dynal, Germany). Thereafter, lymphoma cells were further purified by negative depletion of non-neoplastic bystander B cells using beads coated with antibodies to heavy and light chains not expressed by the lymphoma, including IgD. The purity of lymphocyte cell suspensions obtained after magnetic immunobeads depletion was between 97.5 and 99.7% B cells, as calculated by FACScan analysis.

Purified normal and tumor B cells were cultured in RPMI1640 supplemented with 1% gentamycin, 10% FCS, and 50 $\mu$g/ml transferrin (Sigma, St. Louis). For activation through CD40 antigen, B cells were cultured in the presence of 0.1 $\mu$g/ml anti-CD40 mAb 89 (J. Banchereau, Dardilly, France) presented by a mouse Ltk$^-$ cell line stably expressing CDw32 according to the experimental procedure described previously (Banchereau and Rousset, 1991). Recombinant cytokines were added at the onset of culture. DNA synthesis was determined as measured by [3H] TdR incorporation as described (Greiner et al., 1994b).

### Tumor Immunoglobulin and $\alpha$-Idiotypic Antibodies

MALT-type lymphoma immunoglobulins were produced by fusing lymphoma cells with the heteromyeloma NSO and selected by corresponding to the lymphoma immunoglobulin as described recently (Greiner et al., 1994b). Monoclonal anti-idiotypic antibodies were produced by immunizing mice with purified tumor immunoglobulin. Mouse immunoglobulin-secreting hybridomas were cloned by limiting dilution and selected by exhibiting a restricted specificity in immunohistochemistry, Western-blot, and competitive ELISA for the tumor antigen receptor and not for controls (Greiner et al., 1991).

### Flow Cytometry

Flow cytometric analysis was performed on a FACScan (Becton-Dickinson) with an Argon ion laser tuned at 488 nm using LYSIS II for data acquisition and analysis using triple immunostaining with directly conjugated antibodies: CD19 (HD 37, sigma); kappa (Dako, Hamburg); lambda (Dako); CD3 (UCHT-1, Sigma); CD14 (Leu-M3; Becton-Dickinson); CD40 (mAb89; Banchereau and Rousset, 1991).

### ELISA

Supernatants were assayed in ELISA as described recently (Greiner et al., 1994a) using microtiter plates (Falcon, FRG) coated with goat anti-human heavy-chain (Dako). After blocking with 0.5% BSA, undiluted samples were added for 2 h at 37°C. Finally, the plates were developed with rabbit anti-human heavy-chain HRP conjugate (Dako) and orthophenyl-diamine with hydrogen peroxide in citrate phosphate buffer and read at 490 nm in an automatic ELISA reader (Merlin, FRG). The sensitivity of the assay was < 10 ng/ml.

### RNA Extraction

All tumor tissue blocks were snap-frozen in liquid nitrogen and stored at −70°C until extraction of RNA. Total RNA was prepared (TRIzol reagent; Life Technology, Paisley, UK) from 20 sections of about 10 $\mu$m from the frozen tumor tissue blocks. Integrity of RNA was controlled by electrophoresis through a 2% formaldehyde-agarose gel and the yield of RNA was quantitated by measuring the optical density. To check for carryover of material during the isolation step, extraction buffer without tissue was used as a negative control.

### cDNA Synthesis

First-strand synthesis was performed with 1 $\mu$g of total cellular RNA. RNA, 2 $\mu$g of dT-15 primer, and DEPC-treated water to give a final volume of 8 $\mu$l were incubated for 10 min at 65°C. After chilling on ice, a master mix consisting of dNTPs and dithiothereitol (final concentrations of 1 mmol/l each and 10

mmol/l, respectively), 25 U of recombinant RNAse inhibitor (Promega, Heidelberg), RT-buffer and 200 U of moloney-murine leukemia virus reverse transcriptase (GIBCO BRL) were added to a final reaction volume of 25 $\mu$l. After 70 min of incubation at 37°C, the samples were heated to 98°C for 4 min. The efficiency of cDNA synthesis was estimated by PCR with glyceraldehyde-3-phosphate dehydrogenase (GAPDH)-specific primers.

### Sequence Analysis of MALT Lymphoma VH Genes

The nucleotide sequences of the immunoglobulin VH genes were analyzed using tumor-specific primers and compared with the germ line sequence as described previously (Qin et al., 1995).

### Semiquantitative Polymerase Chain Reaction

For gross quantitation, we used GAPDH-specific expression as an external standard, which was amplified in separate reactions. Thirty-three cycles of amplification for each sample were carried out in a DNA thermal cycler (Perkin-Elmer Centus, Emeryville, CA) and aliquots were taken after 21, 23 and 25 cycles. Each cycle of amplification consisted of 30 sec of denaturation at 94°C, 30 sec of annealing at 60°C, and 45 sec of extension at 72°C. The aliquots were subjected to electrophoresis and the amount of amplification estimated by comparing the signal intensity. After adjusting the cDNA amount in each sample to generate an equal result in GAPDH amplification, a CD40L-specific PCR was carried out (Graf et al., 1992). Thirty-three cycles of amplification were performed to determine the relative amount of CD40L specific mRNA. The amplification conditions for CD40L were 20 sec of denaturation at 94°C, 30 sec of annealing at 60°C, and a 20-second extension at 72°C. To ensure amplification within the linear range, aliquots were taken after different cycles.

### *Acknowledgements*

This work was supported by the Wilhelm-Sander Stiftung, grant 94.025.2 and the Deutsche Forschungsgemeinschaft, DFG grant Mu 579/3-2. We would like to thank Christa Amrehn and Maria Reichert for expert technical assistance.

### *References*

Arpin C., Dechanet J., van Kooten C., Merville P., Grouard G., Briere F., Banchereau J., and Liu Y.J. (1995). Generation of memory B cells and plasma cells in vitro. Science **268**: 720-722.

Banchereau J., Bazan F., Blanchard D., Briere F., Galizzi J.P., van Kooten C., Liu Y.J., Rousset F., and Saeland S. (1994). The CD40 antigen and its ligand. Annu. Rev. Immunol. **12**: 881-922.

Banchereau J., and Rousset F. (1991). Growing human B lymphocytes in the CD40 system. Nature **353**: 678-679.

Briere F., Servet Delprat C., Bridon J.M., Saint Remy J.M., and Banchereau J. (1994). Human interleukin 10 induces naive surface immunoglobulin D+ (sIgD+) B cells to secrete IgG1 and IgG3. J. Exp. Med. **179**: 757-762.

Burdin N., van Kooten C., Galibert L., Abrams J.S., Wijdenes J., Banchereau J., and Rousset F. (1995). Endogenous IL-6 and IL-10 contribute to the differentiation of CD40-activated human B lymphocytes. J. Immunol. **154**: 2533-2544.

Dersimonian H., Schwartz R.S., Barrett K.J., and Stollar B.D. (1987). Relationship of human variable region heavy chain germ-line genes to genes encoding anti-DNA autoantibodies. J. Immunol. **139**: 2496-2501.

Dighiero G. (1992). Autoantibody activity and V gene usage by B-cell malignancies. Leuk. Lymphoma **8**: 345-351.

Dunn Walters D.K., Isaacson P. G., and Spencer J. (1995). Analysis of mutations in immunoglobulin heavy chain variable region genes of microdissected marginal zone (MGZ) B cells suggests that the MGZ of human spleen is a reservoir of memory B cells. J. Exp. Med. **182**: 559-566.

Fluckiger A.C., Garrone P., Durand I., Galizzi J.P., and Banchereau J. (1993). Interleukin 10 (IL-10) upregulates functional high affinity IL-2 receptors on normal and leukemic B lymphocytes. J. Exp. Med. **178**: 1473-1481.

Funakoshi S., Longo D.L., Beckwith M., Conley D.K., Tsarfaty G., Tsarfaty I., Armitage R.J., Fanslow W.C., Spriggs M.K., and Murphy W.J. (1994). Inhibition of human B-cell lymphoma growth by CD40 stimulation. Blood **83**: 2787-2794.

Galibert L., Burdin N., de Saint Vis B., Garrone P., van Kooten C., Banchereau J., and Rousset F. (1996). CD40 and B cell antigen receptor dual triggering of resting B lymphocytes turns on a partial germinal center phenotype. J. Exp. Med. **183**: 77-85.

Graf D., Korthauer U., Mages H.W., Senger G., and Kroczek R.A. (1992). Cloning of TRAP, a ligand for CD40 on human T cells. Eur. J. Immunol. **22**: 3191-3194.

Greiner A., Kirchner T., Ott G., Marx A., Fischbach W., and Müller-Hermelink H.K. (In Press) Occurrence of multiple lymphoepithelioma-like carcinomas and MALT-type lymphoma in the stomach: Detection of EBV in carcinomas but not in lymphoma. Histopathology

Greiner A., Marx A., Heesemann J., Leebmann J., Schmausser B., and Müller-Hermelink H.K. (1994a). Idiotype identity in a MALT-type lymphoma and B cells in Helicobacter pylori associated chronic gastritis. Lab. Invest. **70**: 572-578.

Greiner A., Marx A., Schmausser B., and Müller-Hermelink H.K. (1994b). The pivotal role of the immunoglobulin receptor of tumor cells from B cell lymphomas of mucosa associated lymphoid tissue (MALT). Adv. Exp. Med. Biol. **355**: 189-193.

Greiner A., and Müller-Hermelink H.K. (1996). Recent advances in gastric extranodal B-cell lymphoma. Curr. Diagn. Pathol. **3**: 91-98.

Greiner A., Vollmers H.P., and Müller-Hermelink H.K. (1991). Immunohistochemical characterization of a monoclonal idiotype and anti-idiotype antibody of an IgA-expressing MALT-type lymphoma. In Monoclonal Gammapathies III. Clinical Significance and Basic Mechanism, Radl J., and van Camp, B., Eds. (Leiden, Netherlands: EURAGE), pp. 187-190.

Han S., Hathcock K., Zheng B., Kepler T.B. Hodes R., and Kelsoe G. (1995). Cellular interaction in germinal centers. Roles of CD40 ligand and B7-2 in established germinal centers. J. Immunol. **155**: 556-567.

Harris N.L., Jaffe E.S., Stein H., Banks P.M., Chan J.K., Cleary M.L., Delsol G., De Wolf Peeters C., Falini B., Gatter K.C., and et al. (1994). A revised European-American classification of lymphoid neoplasms: a proposal from the International Lymphoma Study Group. Blood **84**: 1361-1392.

Hussell T., Isaacson P.G., Crabtree J.E., Dogan A., and Spencer J. (1993). Immunoglobulin specificity of low grade B cell gastrointestinal lymphoma of mucosa-associated lymphoid tissue (MALT) type. Am. J. Pathol. **142**: 285-292.

Isaacson P.G. (1994). Gastrointestinal lymphoma. Hum. Pathol. **25**: 1020-1029.

Isaacson P.G., and Spencer J. (1987). Malignant lymphoma of mucosa-associated lymphoid tissue. Histopathology **11**: 445-462.

Kobayashi R., Rassenti L.Z., Meisenholder G., Carson D.A., and Kipps T.J. (1993). Autoantigen inhibits apoptosis of a human B cell leukemia that produces pathogenic rheumatoid factor. J. Immunol. **151**: 7273-7283.

Marin M.C., Hsu B., Stephens L.C., Brisbay S., and McDonnell T.J. (1995). The functional basis of *c-myc* and *bcl*-2 complementation during multistep lymphomagenesis in vivo. Exp. Cell Res. **217**: 240-247.

Müller-Hermelink H.K., Ott G., Ott M., and Greiner A. (1995). Pathology and pathogenesis of extranodal lymphomas in the gastrointestinal tract. Schweiz. Rundsch. Med. Prax. **84**: 1416-1422.

Ohno T., Oka K., Yamaguchi M., Kita K., and Shirakawa S. (1995). Frequent expression of shared idiotypes in mantle cell lymphoma and extranodal small lymphocytic/non-mantle cell diffuse small cleaved lymphoma. Leukemia **9**: 1935-1939.

Olee T., Yang P.M., Siminovitch K.A., Olsen N.J., Hillson J., Wu J., Kozin F., Carson D.A., and Chen P.P. (1991). Molecular basis of an autoantibody-associated restriction fragment length polymorphism that confers susceptibility to autoimmune diseases. J. Clin. Invest. **88**: 193-203.

Ott G., Kirchner T., Seidl S., and Müller-Hermelink H.K. (1993). Primary gastric lymphoma is rarely associated with Epstein-Barr virus. Virchows Arch. B. Cell Pathol. Incl. Mol. Pathol. **64**: 287-291.

Qin Y., Greiner A., Trunk M.J., Schmausser B., Ott M.M., and Müller-Hermelink H.K. (1995). Somatic hypermutation in low-grade mucosa-associated lymphoid tissue-type B-cell lymphoma. Blood **86**: 3528-3534.

Roblick U., Ott G., Kalla J., Hahn U., Ott M.M., and Müller-Hermelink H.K. (1993). Nachweis numerischer Chromosomenabberationen in primären gastralen Non-Hodgkin-Lymphomen durch in-situ-Hybridisierung mit centromerspezifischen DNA-Proben am Gefrierschnitt. Verh. Dtsch. Ges. Pathol. **77**: 487.

Rudders R.A., Levin A., Jespersen D., Zacks J., Delellis R., Ranger A., and Krontiris T. (1992). Crossreacting human lymphoma idiotypes. Blood **80**: 1039-1044.

Sanz I., Dang H., Takei M., Talal N., and Capra J.D. (1989). VH sequence of a human anti-Sm autoantibody. Evidence that autoantibodies can be unmutated copies of germline genes. J. Immunol. **142**: 883-887.

Schmid I., Krall W.J., Uittenbogaart C.H., Braun J., and Giorgi J.V. (1992). Dead cell discrimination with 7-amino-actinomycin D in combination with dual color immunofluorescence in single laser flow cytometry. Cytometry **13**: 204-208.

Schroder A.E., Greiner A., Seyfert C., and Berek C. (1996). Differentiation of B cells in the nonlymphoid tissue of the synovial membrane of patients with rheumatoid arthritis. Proc. Natl. Acad. Sci. USA **93**: 221-225.

Schroeder H.W. Jr., and Dighiero G. (1994). The pathogenesis of chronic lymphocytic leukemia: Analysis of the antibody repertoire. Immunol. Today **15**: 288-294.

Wotherspoon A.C., Finn T.M., and Isaacson P.G. (1995). Trisomy 3 in low-grade B-cell lymphomas of mucosa-associated lymphoid tissue. Blood **85**: 2000-2004.

